# Increased glucose metabolism in *Arid5b*^*−/−*^ skeletal muscle is associated with the down-regulation of TBC1 domain family member 1 (TBC1D1)

**DOI:** 10.1186/s40659-020-00313-3

**Published:** 2020-10-06

**Authors:** Yuri Okazaki, Jennifer Murray, Ali Ehsani, Jessica Clark, Robert H. Whitson, Lisa Hirose, Noriyuki Yanaka, Keiichi Itakura

**Affiliations:** 1 Department of Molecular and Cellular Biology, Beckman Research Institute, City of Hope, Duarte, CA USA; 2 Department of Molecular and Applied Bioscience, Graduate School of Biosphere Science, Hiroshima University, Higashi-Hiroshima, Hiroshima Japan; 3 Department of Central Research Institute, Wakunaga Pharmaceutical Co., Ltd., Akitakata, Hiroshima Japan

**Keywords:** *Arid5b*, Glucose metabolism, TBC1D1, GLUT4 translocation

## Abstract

**Background:**

Skeletal muscle has an important role in regulating whole-body energy homeostasis, and energy production depends on the efficient function of mitochondria. We demonstrated previously that AT-rich interactive domain 5b (*Arid5b*) knockout (*Arid5b*^*−/−*^) mice were lean and resistant to high-fat diet (HFD)-induced obesity. While a potential role of *Arid5b* in energy metabolism has been suggested in adipocytes and hepatocytes, the role of *Arid5b* in skeletal muscle metabolism has not been studied. Therefore, we investigated whether energy metabolism is altered in *Arid5b*^*−/−*^ skeletal muscle.

**Results:**

*Arid5b*^*−/−*^ skeletal muscles showed increased basal glucose uptake, glycogen content, glucose oxidation and ATP content. Additionally, glucose clearance and oxygen consumption were upregulated in *Arid5b*^*−/−*^ mice. The expression of glucose transporter 1 (GLUT1) and 4 (GLUT4) in the gastrocnemius (GC) muscle remained unchanged. Intriguingly, the expression of TBC domain family member 1 (TBC1D1), which negatively regulates GLUT4 translocation to the plasma membrane, was suppressed in *Arid5b*^*−/−*^ skeletal muscle. Coimmunofluorescence staining of the GC muscle sections for GLUT4 and dystrophin revealed increased GLUT4 localization at the plasma membrane in *Arid5b*^*−/−*^ muscle.

**Conclusions:**

The current study showed that the knockout of *Arid5b* enhanced glucose metabolism through the downregulation of TBC1D1 and increased GLUT4 membrane translocation in skeletal muscle.

**Electronic supplementary material:**

The online version of this article (10.1186/s40659-020-00313-3) contains supplementary material, which is available to authorized users.

## Background

AT-rich interactive domain 5B (ARID5B) is a member of the AT-rich interactive domain (ARID) transcriptional factor family, which consists of seven subfamilies, ARID1 through ARID5, Jumonji ARID 1 (JARID1) and JARID2. The ARID proteins play diverse roles in cell proliferation, cell cycle control, differentiation, senescence and development [1]. Several previous reports have shown that *Arid5b* regulates cell differentiation of the mesenchymal stem cell (MSC) lineage; in vitro studies demonstrated that adipogenesis was significantly suppressed in 3T3-L1 preadipocytes by knockdown of *Arid5b* and in mouse embryonic fibroblasts (MEFs) isolated from *Arid5b* knockout (*Arid5b*^*−/−*^) mice [2]. In addition, recent studies have shown that *Arid5b* promotes differentiation in skeletal muscle satellite cells (SCs) and chondrocytes; Murray et al*.* demonstrated that the deletion of *Arid5b* in SCs impaired prostaglandin (PG) I_2_ (PGI_2_) production through the downregulation of cyclooxygenase-1 (COX-1) and PGI_2_ synthase (PTGIS). Decreased production of PGI_2_ increased cell migration and inhibited myotube formation and fusion in *Arid5b*^*−/−*^ SCs, which were rescued by supplementation with the PGI_2_ analog, iloprost [3]. Hata et al*.* identified ARID5B as a co-regulator of SRY-box transcription factor 9 (SOX9) that recruits plant homeodomain (PHD) finger protein 2 (PHF2), a histone demethylase, to the promoters of Sox9-target genes, such as collagen type II alpha 1 chain (*Col2a1*) and *aggrecan*. The authors showed that the ARID5B-PHF2 complex removes the dimethylated lysine 9 on histone 3 (H3K9Me2) repression mark and activates transcription of these target genes to facilitate chondrogenesis in mice [4]*.*

Interestingly, we found that *Arid5b*^*−/−*^ mice had reduced white adipose tissue mass relative to *Arid5b* wild-type (*Arid5b*^+*/*+^) mice and were resistant to high-fat diet (HFD)-induced obesity [5]. Additionally, knock-down of *Arid5b* in 3T3-L1 adipocytes activated free fatty acid recycling into cellular triglyceride by increasing both lipolysis and triglyceride synthesis [5, 6]. Moreover, in hepatocytes, it has been demonstrated that ARID5B forms a complex with PHF2 on the promoters of its target genes, phosphoenolpyruvate carboxykinase (*Pepck)* and glucose-6 phosphatase (*G6Pase*), in response to protein kinase A (PKA) activation. The ARID5B-PHF2 complex activates transcription of these genes by removing the repressive H3K9Me2 mark from their promoters [7]. These findings suggest *Arid5b* may play a role in energy metabolism in various cell types.

Skeletal muscle is one of the main tissues utilizing glucose and fatty acids for ATP generation and maintains whole-body energy homeostasis by responding to energy demands and nutrient availability [8]. Skeletal muscle takes up glucose from the blood in response to insulin stimuli and maintains blood glucose levels [9]. In fact, impairment of glucose transport into skeletal muscle due to insulin resistance in type II diabetes is a serious condition that eventually disrupts functions of other tissues [10]. In addition, the activity of mitochondrial oxidative metabolism determines the efficiency of energy production from both glucose and fatty acids [8]. Since the consequence of *Arid5b* deletion in skeletal muscle metabolism has not been studied, we investigated the potential role of *Arid5b* in skeletal muscle metabolism using *Arid5b*^*−/−*^ mice. In this study, we report that basal glucose uptake and glucose oxidation were enhanced in *Arid5b*^*−/−*^ skeletal muscle and were associated with the downregulation of TBC domain family member 1 (TBC1D1) expression.

## Methods

### Animal studies

*Arid5b*^*−/−*^ mice were established by homologous recombination with a PGK promoter-driven neomycin cassette as described previously [5]. Mice were fed standard chow ad libitum and maintained under controlled light–dark cycles (light cycle 6AM to 6PM). 10 to 21 week-old, standard chow-fed male mice were used for all experiments and euthanized between 11AM-2PM unless otherwise specified. Movements, O_2_ consumption and CO_2_ production, and food intake of mice were recorded using the TSE PhenoMaster V5.9.3 (2016–5420) at the Comprehensive Metabolic Phenotyping Core at Beckman Research Institute (Duarte, California). Mice were individually placed in the apparatus for at least 16 h in order to acclimate, and then movements, O_2_ consumption and CO_2_ production, and food intake of mice were measured every thirty minutes over the next 48 h. Four different skeletal muscles (gastrocnemius muscle [GC], quadriceps [QC], soleus [SoL], extensor digitorum longus [EDL]) were isolated from mice. Whole SoL or EDL muscles were used for oxidation assays and glucose uptake assays.

### Quantitative real-time PCR (qRT-PCR)

Skeletal muscle tissue powder (30 mg) was homogenized with the POLYTRON ® homogenizer (Kinematica, #PT2100) in 700 μL of QIAzol® Lysis Reagent. Total RNA was purified from the homogenate using the miRNeasy Mini Kit (Qiagen) according to the manufacturer’s instructions. Reverse transcription was performed using 0.75 μg RNA following the instructions of the iScript™ cDNA synthesis kit (Bio-Rad). Experimental transcript levels were analyzed using iQTM SYBR Green Supermix (Bio-Rad) on the CFX96™ Real-Time System. All qRT-PCR data was normalized to ribosomal protein L13a *(Rpl13a)* expression analyzed in separate reactions. Primer sequences are listed in Table 1.

**Table 1:** Primers for qRT-PCR

Gene symbol	Forward (5′–3′)	Reverse (5′–3′)
*Arid5b*	AGAAAAACGCCCATCGAGC	CTCCCAGGATTACCACCTAAC
*Rpl13a*	TGCTGCTCTCAAGGTTGTTC	TTTCCTTCCGTTTCTCCTCC

### Western blotting analysis

Whole-tissue protein lysate was prepared from powdered GC muscle by sonication with the Q700 sonicater (QSONICA, ice-water bath mode) in Tris-NaCl-Sucrose (TNS) buffer (20 mM Tris–HCl, pH7.4/50 mM NaCl/250 mM sucrose) supplemented with 20 mM NaF, 1 mM dithiothreitol (DTT), 1 mM phenylmethylsulfonyl fluoride (PMSF, Thermo Fisher Scientific) and 1 × Halt® Protease and Phosphatase Inhibitor Cocktail (100 × stock solution, Thermo Fisher Scientific). 200 μL of TNS buffer was used for 10 mg of skeletal muscle tissue. Sonication was performed six times at amplitude 35 for 1 min with intervals of 1 min on ice. After the sonication, Triton X-100 was added to the homogenate to give 1% final concentration, and the lysates were incubated at 4 °C for one hour on a tube rotator. The lysates were briefly centrifuged at 10,000 × *g* at 4 °C. Protein concentration of each lysate was determined with the Pierce™ BCA assay kit (Thermo Fisher Scientific, #23225). SDS-PAGE was carried out using approximately 10–60 μg of protein lysate with Criterion™ TGX™ precast gels (Bio-Rad), and protein was transferred to PVDF membranes (Bio-Rad) using the Trans-Blot Turbo System (Bio-Rad). Specific protein detection was performed on the membranes using anti-phospho- AMP-activated protein kinase (AMPK) α1/2 (Thr172) antibody (Cell Signaling, #2535), anti-AMPKα antibody (Cell Signaling, #2603), anti-peroxisome proliferator-activated receptor gamma coactivator-1 alpha (PGC-1α) antibody (Abcam, #ab54481), anti-myosin heavy chain 7 (MYH7) antibody (Sigma, #M8421), anti-myosin heavy chain 1 (MYH1) antibody (Thermo Fisher Scientific, #PA5-31466), anti-glucose transporter 4 (GLUT4) (Cell Signaling, #2213), anti-glucose transporter 1 (GLUT1) (Novus Biologicals, #NB110-39113), anti-TBC1D1 (Cell Signaling, #4629), anti-TBC domain family member 4 (TBC1D4) (Cell Signaling, #2670), anti-heat shock protein 70 (HSP70) antibody (Cell Signaling, #4872), anti-heat shock protein 90 (HSP90) antibody (Cell Signaling, #4877), and anti-vinculin (Cell Signaling, #13901). Western BLoT Immuno Booster (Takara, #T7111A) was used to incubate antibodies for the detection of GLUT4, TBC1D1, and TBC1D4. Proteins were detected with Amersham ECL Prime (GE Healthcare), HyGLO™ Chemiluminescent detection reagent (Denville Scientific Inc.), or Prosignal Dura ECL Reagent (Prometheus, #84–834) after secondary antibody incubation.

### Glucose and fatty acid oxidation assays

Two different skeletal muscles, SoL and EDL, were isolated from non-fasted *Arid5b*^+*/*+^ mice and *Arid5b*^*−/−*^ mice and used for glucose oxidation and fatty acid oxidation assays. For glucose oxidation assays, isolated skeletal muscles were pre-incubated in Krebs–Henseleit Bicarbonate (KHB) buffer (116 mM NaCl, 4.6 mM KCl, 1.16 mM KH_2_PO_4_, 25.3 mM NaHCO_3_, 2.5 mM CaCl_2_, 1.16 mM MgSO_4_) supplemented with 2 mM glucose, 38 mM mannitol, and 2% fatty-acid free bovine serum albumin (BSA) (hereafter referred to as a glucose oxidation assay buffer) and placed on ice and transferred to 37 °C with 5% CO_2_ for 40 min before the assay. Glucose D- [^14^C] (uniformly labeled, American Radiolabeled Chemicals, Inc., #ARC0122E, 1 mCi/mL) and 50 mM palmitic acid were added into KHB buffer to give a final concentration of 1 μCi/mL of glucose D- [^14^C] and 50 μM palmitic acid. Skeletal muscles were incubated in 500 μL of the buffer in scintillation vials (Research Products International Corp, #121000CA) closed with a rubber stopper (Kimble Chase, #882310–0000) and parafilm for one hour at 37 °C under 5% CO_2_ with 200 μL of benzethonium hydroxide (Sigma, B2156) as an acceptor of released ^14^CO_2_ from skeletal muscle during incubation. At the end of the incubation, 100 μL of 60% perchloric acid (Sigma. #311413) was added per vial to stop the reaction. The vials were covered with new parafilm and stored at 4 °C overnight. Benzethonium hydroxide was transferred into a new scintillation vial and shaken vigorously with 10 mL of Ecoscint™ A (National Diagnostics, #LS-273). Radioactivity of [^14^C] was measured for 1 min per sample with the Beckman Liquid Scintillation Counter™ (#LS6500).

For fatty acid oxidation assays, isolated skeletal muscles were pre-incubated in KHB buffer supplemented with 10 mM HEPES, 5 mM glucose, and 4% fatty-acid free BSA (hereafter referred to as fatty acid oxidation assay buffer) on ice and transferred to 37 °C with 5% CO_2_ for 30 min before the assay. Palmitic acid- [9, 10-^3^H]-BSA complex was prepared before dissection of skeletal muscles by following steps. 1.6 × 10^–6^ μmol of palmitic acid- [9, 10-^3^H] (Moravek, Inc., #MT-845) and 4 μmol of cold palmitic acid were mixed with 3 μmol of KOH and the mixture was dried under nitrogen gas. The dried palmitic acid- [9, 10-^3^H] was complexed to BSA by resuspension in 25% BSA solution to give a final concentration of 1.6 × 10^–6^ mM palmitic acid- [9, 10-^3^H] and 4 mM cold palmitic acid (fatty acid-BSA stock). Fifty microliters of fatty acid-BSA stock was added to 1 mL of fatty acid oxidation assay buffer in round bottom culture tubes (12 × 75 mm, #110428, Globe Scientific Inc.), and skeletal muscle was incubated in the buffer for two hours at 37 °C under 5% CO_2_. After incubation, 400 μL of supernatant was transferred into a new 15 mL tube, and 2 mL of chloroform–methanol (2:1, vol/vol) was added in order to separate ^3^H_2_O from palmitic acid- [9, 10-^3^H] in the supernatant. The solution was vortexed for 10 s and centrifuged briefly at 3000 × *g* at room temperature (RT). 0.8 mL of 2 M KCl-2 M HCl was added to the solution, vortexed again and centrifuged at 3000 × *g* for 5 min at RT. Radioactivity of 0.5 mL of the aqueous phase was measured for [^3^H] for 5 min per sample with the Beckman Liquid Scintillation Counter™ (#LS6500). Skeletal muscles used for glucose and fatty oxidation assay were washed in dH_2_O, placed on aluminum foil and dried at 60 °C for 20–24 h. Dry tissue weights were measured and used for normalization of radioactivity of each sample.

### Quantification of metabolites and enzyme activity

Powdered GC muscles (10–20 mg per assay) were sonicated in the appropriate assay buffer (Q700 ice-water bath sonicator, QSONICA) for six cycles of amplitude 35 for 30 sec followed by 1 min on ice, centrifuged at 10,000 × *g* for 10 min at 4 °C and the lysates were subjected to the following assays: measurement of the metabolites and citrate synthase (CS) activity. Lactate Colorimetric Assay (Biovision, #K607-100), Glycogen Colorimetric Assay (Biovision, #K648-100), and Citrate Synthase Activity Colorimetric Assay (Biovision, #K318-100) were performed according to the manufacturers’ instructions.

### Nucleotide extraction

Powdered GC muscles (10-15 mg) were sonicated in 3% trichloroacetic acid (TCA)/PBS (Q700 ice-water bath sonicator, QSONICA) for six cycles at amplitude 35 for 30 s followed by 1 min on ice and centrifuged at 12,000×*g* for 5 min at 4 °C. The supernatant was transferred into a new ice-cold tube, diluted with an equal volume of ddH_2_O, and pH was adjusted to neutral with 1 M KOH. The amounts of ATP, ADP and AMP in each sample were measured by UV-HPLC in the Analytical Pharmacology Core at City of Hope (Duarte, CA). The pellet after centrifugation was dissolved in 5% sodium dodecyl sulfate (SDS)/ 0.1 N NaOH, and protein concentration was measured by BCA assay (Thermo Fisher Scientific).

### Intraperitoneal glucose tolerance test (IP-GTT) and plasma insulin concentration

The mice were fasted for 7–8 h before the glucose tolerance test was conducted. Whole blood samples were collected from tails of mice before (0 min), and 10, 20, 30, 60 and 120 min after an i.p. injection of glucose (2 mg D-glucose/g body weight) for the measurement of blood glucose levels with a Clarity Diagnostics BG1000 glucose meter. The plasma was prepared by centrifugation at 1,000×*g* for 10 min. The plasma insulin levels were determined using Insulin (mouse, rat) EIA kits (Cayman Chemical, #589501) by following the manufacturers’ instructions. The plasma samples collected during IP-GTT were spiked with 1 ng/mL of insulin standard provided in the kit. The data were analyzed using the analysis workbook (ELISADouble) available in Cayman’s website for the determination of the insulin concentration of standards and the spiked plasma samples. The insulin concentration of the plasma samples was calculated by subtracting the average concentration of 1 ng/mL of insulin standard samples (duplicate) from the spiked plasma samples. The area under the curve (AUC) was calculated from the IP-GTT curve using the trapezoidal rule.

### Glucose uptake assay

We followed the previously published method to measure glucose uptake into skeletal muscle with some modifications [11]. Briefly, mice were fasted for 7–8 h and SoL muscles were isolated for the assay. Muscles were incubated for 40 min at 30 °C (5% CO_2_ + 95% O_2_) in a 24-well plate with 1 mL of the modified KHB buffer: 4.7 mM KCl, 1.2 mM KH_2_PO_4_, 118 mM NaCl, 1.2 mM MgSO_4_, 2.5 mM CaCl_2_, 15 mM NaHCO_3_, 1% fatty acid free BSA, 10 mM HEPES, and 1 mM glucose. After the pre-incubation, muscles were placed in 2 mL of KHB containing 1 mM glucose and radiolabelled 2-deoxyglucose- [^3^H] (2-DG) (1.5 μCi/mL) with (150 nM) or without insulin (Gibco, #12585–014). We followed the methods in [11] for the steps of washing (1 mL of KHB containing 1 mM glucose) and weighing muscles and determination of glucose uptake by counting the radioactivity of [^3^H] using a scintillation counter (Beckman Counter™, #LS6500).

### Coimmunofluorescence staining

GC muscle was frozen immediately after isolation in isopentane cooled with liquid nitrogen. GC muscle was cryosectioned at a thickness of 10 µm and affixed to Tissue Path Superfrost Plus Gold microscope slides (Fisher Scientific). Sections were fixed in 4% formaldehyde in PBS and then permeabilized in 0.15% Triton-X100 (Fisher Scientific) in PBS. Antibodies to GLUT4 (#MA5-17176, 1:500, Thermo Fisher Scientific) and dystrophin (#ab15277, 1:400, Abcam) were applied simultaneously to the sections for 2 h at RT. After incubation with primary antibodies, sections were washed 3 times in PBS for 5 min each time. Secondary antibodies were applied to the sections for 45 min at RT at a dilution of 1:500. For GLUT4 we used goat anti-mouse Alexa Fluor 488 (Thermo Fisher Scientific), and for dystrophin we used goat anti-rabbit Alexa Fluor 555 (Thermo Fisher Scientific) secondary antibodies. Sections were then washed 3 times in PBS for 5 min each time and mounted in ProLong Glass Antifade Mountant (Thermo Fisher Scientific). Images (n > 10 per mouse) were taken on the Zeiss LSM 880 inverted confocal microscope (Carl Zeiss, Inc.). All the images were captured with the same laser intensities. The Pearson’s correlation coefficient for quantitating colocalization of GLUT4 and dystrophin was calculated for each image using Zen Black software (Carl Zeiss, Inc.).

### Statistical analysis

Data were expressed as the means ± SD. Statistical significance between *Arid5b*^+*/*+^ mice and age-matched *Arid5b*^−/−^ mice was assessed by Student’s *t* test (unpaired, two tailed). A *P* value of < 0.05 was considered significant.

## Results

### Deletion of *Arid5b* in mice did not alter normalized skeletal muscle weight

**Fig. 1:**
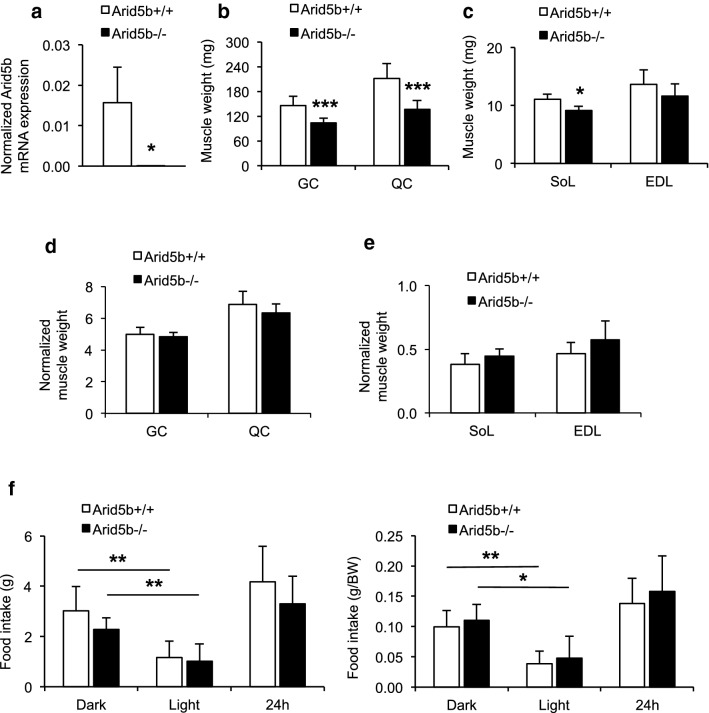
Analyses of body composition and food intake of *Arid5b*^+*/*+^ and *Arid5b*^*−/−*^ mice. **a** *Arid5b* expression in GC muscle. Total RNA was isolated from GC muscle and qRT-PCR analysis was performed. Gene expression was normalized to *Rpl13a* mRNA expression. *Arid5b*^+*/*+^, n = 7; *Arid5b*^*−/−*^, n = 3. *,  *P* < 0.05. **b**, **c** Absolute muscle weights in *Arid5b*^+*/*+^ and *Arid5b*^*−/−*^ mice. GC, QC, SoL and EDL muscles were dissected from male *Arid5b*^+*/*+^ mice and *Arid5b*^*−/−*^ mice, and the weight was measured. **d**, **e** Normalized skeletal muscle weight was determined by taking the ratio of each skeletal muscle weight to the body weight of the mice. Results are presented as the means ± SD. *, *P* < 0.05; ***, *P* < 0.001. GC and QC: *Arid5b*^+*/*+^ mice, n = 18; *Arid5b*^*−/−*^ mice, n = 9. SoL and EDL: *Arid5b*^+*/*+^ mice, n = 3; *Arid5b*^*−/−*^ mice, n = 4. **f** Absolute food intake and normalized food intake relative to body weight (BW) in each genotype (n = 5 per group). Data are presented as the means ± SD. *, *P* < 0.05; **, *P* < 0.01 (dark period *vs* light period)

To confirm that the *Arid5b* gene is knocked out in skeletal muscle, its mRNA expression was examined in GC muscle with a primer set designed in the deletion region of *Arid5b* gene (Table. 1). *Arid5b* mRNA expression was undetectable by qRT-PCR in *Arid5b*^*−/−*^ GC muscle (Fig. 1a). We previously reported that the body weight of the *Arid5b*^*−/−*^ mice was significantly reduced [5]. Weights of GC, QC, and SoL were significantly decreased in *Arid5b*^*−/−*^ mice compared to *Arid5b*^+*/*+^ mice because of the reduction in body weight (Fig. 1b, c), while the weight of EDL did not change. However, the normalized weight of individual skeletal muscles was similar between the genotypes (Fig. 1d, e). Notably, the absolute food intake and normalized food intake relative to body weight did not differ between the genotypes (Fig. 1f).

### Glucose oxidation was enhanced in *Arid5b*^*−/−*^ skeletal muscle

**Fig. 2:**
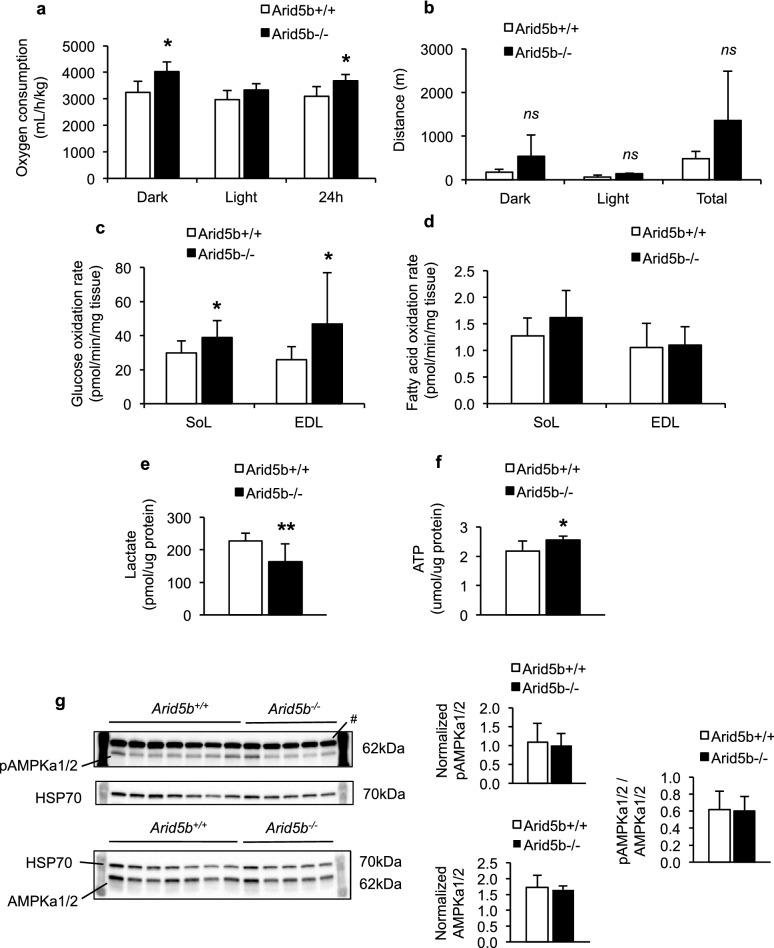
Glucose oxidation was enhanced in *Arid5b*^*−/−*^ skeletal muscles. **a** Oxygen consumption rates were measured and normalized to body weight (n = 5). *, *P* < 0.05. **b** Average distance traveled in two dark vs light periods of 24 h and total distance traveled in 48 h (n = 5) is shown. **c** Glucose oxidation rates and **d** fatty acid oxidation rates were measured in isolated skeletal muscles from non-fasted mice. *Arid5b*^+*/*+^ mice (n = 13–15) and *Arid5b*^*−/−*^ mice (n = 5–6). *, *P* < 0.05. **e** Lactate levels and **f** ATP content was analyzed in GC muscles. *Arid5b*^+*/*+^ mice (n = 10) and *Arid5b*^*−/−*^ mice (n = 5). *, *P* < 0.05; **, *P* < 0.01. **g** The phosphorylation levels of AMPKα1/2 at Thr172 and protein levels of total AMPKαα1/2 were normalized to loading control (HSP70), and the ratio of pAMPKα1/2 (Thr172) to total AMPKα1/2 was calculated. Values represent means ± SD. *Arid5b*^+*/*+^ mice, n = 7; *Arid5b*^*−/−*^ mice, n = 5. #, HSP70 was detected in the same membrane as pAMPKα1/2 (Thr172)

To characterize the metabolic phenotype of skeletal muscle in *Arid5b*^*−/−*^ mice, we first measured oxygen consumption and found that it was significantly increased in *Arid5b*^*−/−*^ mice compared to *Arid5b*^+*/*+^ mice (Fig. 2a). We also measured the basal locomotor activity of the mice and found no significant change in total distance traveled (Fig. 2b). Since oxygen consumption was increased in *Arid5b*^*−/−*^ mice, we investigated the substrate oxidation rates in mitochondria using the isolated skeletal muscles, SoL and EDL. In both types of skeletal muscles, the glucose oxidation rate was significantly elevated by 1.3- to 1.8-fold in *Arid5b*^*−/−*^ skeletal muscle compared to *Arid5b*^+*/*+^ skeletal muscle (Fig. 2c). The fatty acid oxidation rate did not change in the SoL or EDL of *Arid5b*^*−/−*^ mice relative to *Arid5b*^+*/*+^ mice (Fig. 2d). We analyzed lactate content as an end product of anaerobic glycolysis and ATP content in GC muscle and found that lactate content was decreased in *Arid5b*^*−/−*^ GC muscle (Fig. 2e) while ATP content was increased (Fig. 2f). The amount of AMP and ADP and the ratio of AMP/ATP did not change (Additional file 1: Fig. S1). Additionally, the expression and phosphorylation levels of AMPKα1/2 (Thr172) did not change (Fig. 2g). Therefore, the changes in the glucose metabolism in the GC skeletal muscle were not due to AMPK activation.

Next we investigated the potential cause of the increased glucose oxidation in skeletal muscle. Previous studies have shown that enhanced oxidative metabolism in skeletal muscle and other cell types is associated with highly fused mitochondria [12, 13]. Therefore, we analyzed the morphology of mitochondria and the integrity of sarcomeres in myofibers using TEM imaging. There were no noticeable changes in the sarcomere arrangement or cristae morphology of mitochondria in white GC muscle between the two genotypes (data not shown). It has been shown that multiple mitochondrial fusion and fission factors, such as mitofusin 1 (MFN1), mitofusin 2 (MFN2), optic atrophy 1 (OPA1) and dynamin-related protein 1 (DRP1), regulate mitochondrial morphology [14, 15]; however, we did not observe significant alteration in the expression levels of MFN1, MFN2, OPA1 and DRP1 in *Arid5b*^*−/−*^ GC muscle (Additional file 1: Fig. S2). These data suggest that the increase in oxidative metabolism in *Arid5b*^*−/−*^ skeletal muscle was not related to mitochondrial dynamics.

### The expression of PGC-1α and CS activity did not change in *Arid5b*^*−/−*^ GC muscle

**Fig. 3:**
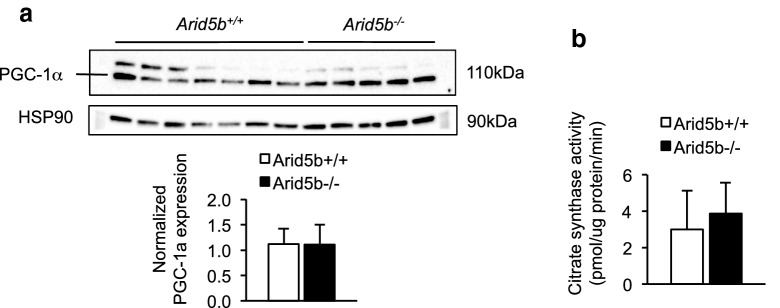
Analyses of genes regulating mitochondrial biogenesis. **a** Western analysis was performed for PGC-1α expression and normalized to HSP90 expression. (bottom) Quantitation of PGC-1α expression was performed. **b** CS activity was analyzed in GC muscle. Data are presented as the means ± SD. *Arid5b*^+*/*+^ mice (n = 7) and *Arid5b*^*−/−*^ mice (n = 5)

To determine if *Arid5b*^*−/−*^ skeletal muscle showed an increase in mitochondrial biogenesis, we analyzed the expression levels of PGC-1α and electron transport chain (ETC) subunits in *Arid5b*^*−/−*^ skeletal muscle and assessed CS activity in *Arid5b*^*−/−*^ skeletal muscle. PGC-1α is a major regulator of mitochondrial biogenesis [16, 17], and CS activity is used as a marker of mitochondrial content [18]. The protein expression of PGC-1α was similar between the genotypes (Fig. 3a); moreover, CS activity did not show a difference between the genotypes (Fig. 3b). The expression of the ETC complexes did not change relative to *Arid5b*^+*/*+^ GC muscle (Additional file 1: Fig. S3). These data indicate that the increased oxygen consumption and glucose oxidation in *Arid5b*^*−/−*^ skeletal muscle are not due to changes in mitochondrial biogenesis.

### The expression levels of myosin heavy chain (Myh) isoforms were not altered in *Arid5b*^*−/−*^ GC muscle

**Fig. 4:**
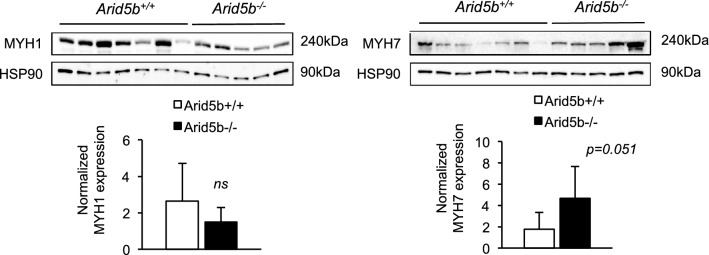
Expression levels of Myh isoforms. Whole lysate was prepared from GC muscle and subjected to immunoblotting. Immunoblot images of MYH7 and MYH1 and the loading control (HSP90) are shown. Quantiation of MYH7 and MYH1 expression was performed, and data are presented as means ± SD (n = 5–7)

To assess whether a fiber-type switch from fast-glycolytic to slow-oxidative could be a potential cause of the increased glucose oxidation in *Arid5b*^*−/−*^ skeletal muscle, we analyzed mRNA expression of the Myh isoforms, *Myh7*, *Myh2*, *Myh1* and *Myh4,* in GC muscle. We found that *Myh1* expression was significantly decreased in *Arid5b*^*−/−*^ GC muscle relative to *Arid5b*^+*/*+^ GC muscle (Additional file 1: Fig. S4). The expression of *Myh7*, *Myh2,* and *Myh4* in *Arid5b*^*−/−*^ GC muscle was comparable to *Arid5b*^+*/*+^ GC muscle (Additional file 1: Fig. S4). At the protein level, the expression of MYH1 was not significantly decreased (Fig. 4). The protein expression of MYH7 was not altered in *Arid5b*^*−/−*^ GC muscle (Fig. 4). Taken together, although the expression of *Myh1* was perturbed at the mRNA level, the myofiber type did not change in *Arid5b*^*−/−*^ GC muscle.

### Whole-body glucose clearance and glucose transport in skeletal muscle were increased in *Arid5b*^*−/*−^ mice

**Fig. 5:**
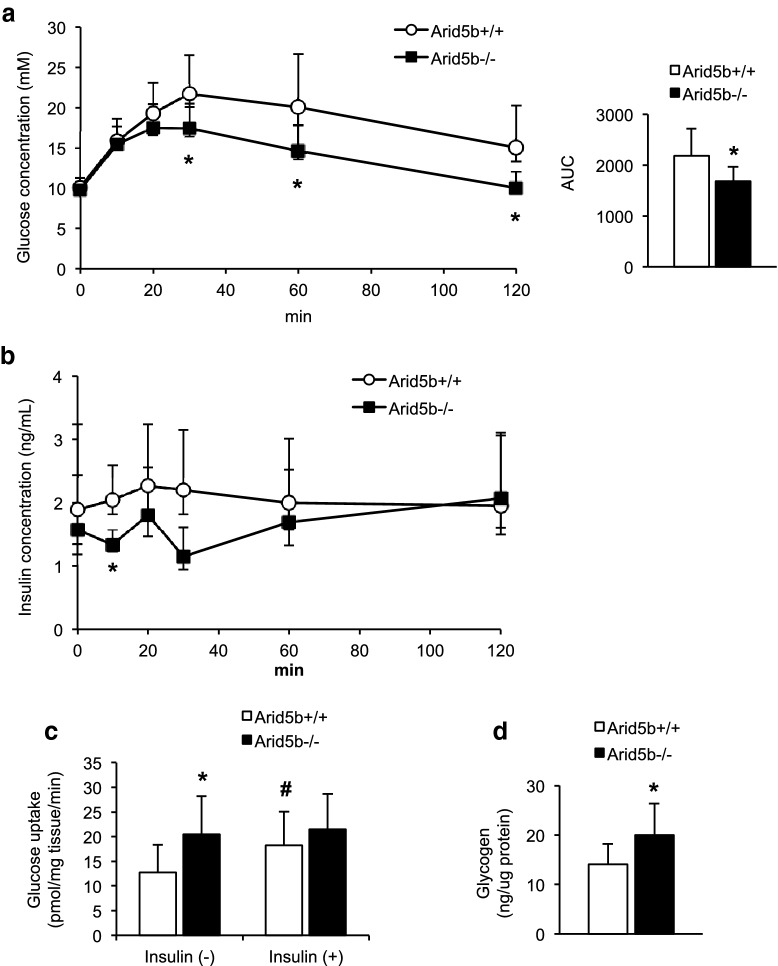
Glucose tolerance test (GTT) and glucose uptake assay. **a** Blood glucose was measured after a 7–8 h fast at baseline (0 min) and 10, 20, 30, 60 and 120 min after an i.p. injection of glucose (left), and AUC was calculated (right) (n = 9). **b** Plasma insulin concentration was measured during the GTT (left, n = 5–6). **c** The rates of basal and insulin-stimulated (150 nM) glucose uptake in SoL were determined with or without insulin 20 min after the addition of 2-DG in KHB containing 1 mM glucose. *Arid5b*^+*/*+^ mice, n = 11–13; *Arid5b*^*−/−*^ mice, n = 8. **d** Glycogen content was quantitated in gastrocnemius muscle. *Arid5b*^+*/*+^ mice, n = 10; *Arid5b*^*−/−*^ mice, n = 5. Results are expressed as the means ± SD. *, *P* < 0.05 *vs Arid5b*^+*/*+^ mice; #, *P* < 0.05 vs insulin (-)

We further characterized systemic glucose metabolism by assessing glucose tolerance in mice and glucose uptake in isolated skeletal muscles. The glucose tolerance test revealed increased glucose clearance in *Arid5b*^*−/−*^ mice relative to *Arid5b*^+*/*+^ mice while insulin concentration significantly decreased at only the 10 min time point in *Arid5b*^*−/−*^ mice (Fig. 5a, b). We observed an increase in basal glucose uptake in SoL isolated from *Arid5b*^*−/−*^ mice, which was consistent with the increased glucose clearance (Fig. 5c). Glucose uptake was significantly stimulated by insulin treatment in SoL from *Arid5b*^+*/*+^ mice as expected, but it was not stimulated in SoL from *Arid5b*^*−/−*^ mice. Because the basal level of glucose uptake in SoL from *Arid5b*^*−/−*^ mice was similar to the insulin-stimulated level in SoL from *Arid5b*^+*/*+^mice, it is possible that uptake in *Arid5b*^*−/−*^ SoL is maximally-stimulated in an insulin-independent manner. Additionally, we measured the amount of glycogen in *Arid5b*^*−/−*^ GC muscle since its levels are associated with the levels of glucose utilization in skeletal muscle [19]. We found that glycogen content was greater in *Arid5b*^*−/−*^ GC muscle compared to *Arid5b*^+*/*+^ GC muscle (Fig. 5d), suggesting increased glucose uptake.

### The expression of TBC1D1 was downregulated and GLUT4 translocation to the plasma membrane was increased in *Arid5b*^*−/−*^ GC muscle

**Fig. 6:**
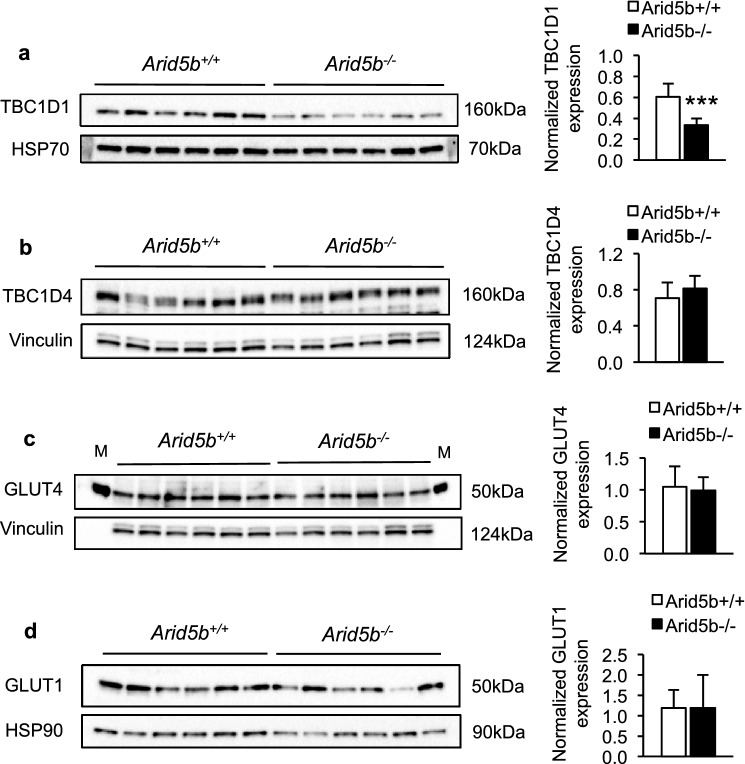
Downregulation of TBC1D1 expression in *Arid5b*^*−/−*^ GC muscles. The expression of TBC1D1 protein (**a**), TBC1D4 protein (**b**) is shown. Protein expression was normalized to the corresponding loading control (HSP90, HSP70 or vinculin), and the data are presented as the means ± SD (n = 6). ***, *P* < 0.001. **c**, **d** The expression of GLUT4 and GLUT1 protein in GC muscles is shown. Quantification of GLUT4 and GLUT1 protein in GC muscles is shown. (bottom) Quantitation of GLUT4 protein expression was performed, and the data are presented as the means ± SD (n = 6)

Since basal glucose uptake was increased in *Arid5b*^*−/−*^ skeletal muscle, we speculated that Akt activation and GLUT4 translocation may be altered in *Arid5b*^*−/−*^ skeletal muscle at basal levels. We first analyzed the protein expression levels of Akt and its phosphorylation at Ser473 by the mammalian target of rapamycin complex 2 (mTORC2) [20]. We found that the levels of expression and phosphorylation at Ser473 of Akt remained similar in *Arid5b*^*−/−*^ GC muscle compared to *Arid5b*^+*/*+^ GC muscle (Additional file 1: Fig. S5), suggesting that the changes in glucose metabolism in *Arid5b*^*−/−*^ muscle are not due to alterations in Akt signaling. Several publications suggest that TBC1D1 and TBC1D4 (also known as AS160) negatively regulate GLUT4 translocation from the intracellular space to the plasma membrane through their Rab-GTPase activity. Since these factors are key regulators of glucose uptake in skeletal muscle [21, 22], we evaluated their mRNA and protein expression levels in *Arid5b*^*−/−*^ GC muscle. Interestingly, protein expression levels of TBC1D1 but not mRNA expression levels were significantly downregulated in *Arid5b*^*−/−*^ GC muscle (Fig. 6a and Additional file 1: Fig. S6a) compared to *Arid5b*^+*/*+^ GC muscle whereas expression of TBC1D4 did not change (Fig. 6b and Additional file 1: Fig. S6b). Since the deletion or downregulation of *Tbc1d1* in mice has been shown to influence the protein expression of GLUT4 [11, 22, 23], we evaluated the basal protein expression of GLUT4 and GLUT1 in *Arid5b*^*−/−*^ GC muscle. However, no significant difference was observed in the expression of either protein between the genotypes (Fig. 6c, d).

**Fig. 7:**
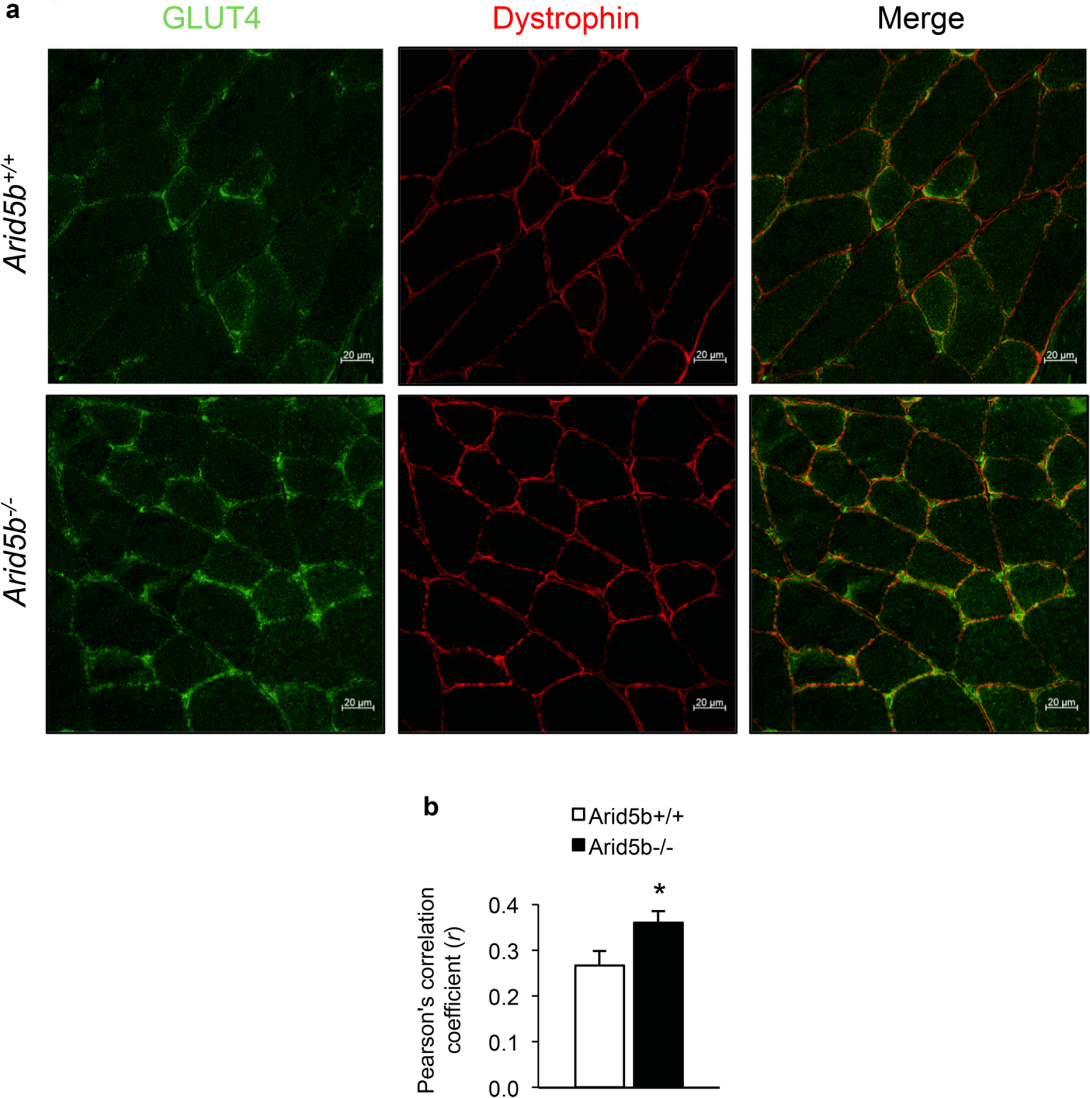
GLUT4 is localized to the plasma membrane in *Arid5b*^*−/−*^ GC muscles. **a** Coimmunofluorescence analysis was performed for GLUT4 (green) and dystrophin (red) in GC muscle sections, and representative confocal images are shown. Merged images show increased colocalization of GLUT4 and dystrophin at the plasma membrane in *Arid5b*^*−/−*^ GC muscles relative to *Arid5b*^+*/*+^ GC muscles. **b** The Pearson’s correlation coefficient quantifying the degree of colocalization was calculated (n > 10 images per mouse, n = 3 mice per genotype). Data are presented as the means ± SD. *, *P* < 0.05

To determine whether there was an increase in GLUT4 membrane translocation in *Arid5b*^*−/−*^ muscle, we performed coimmunofluorescence staining for GLUT4 and dystrophin, a plasma membrane marker, on GC muscle sections from *Arid5b*^+*/*+^ and *Arid5b*^*−/−*^ mice. We observed an increase in GLUT4 translocation to the membrane in *Arid5b*^*−/−*^ GC relative to *Arid5b*^+*/*+^ GC muscle (Fig. 7a). We then calculated the Pearson’s correlation coefficient, which is a commonly used method to quantitate the extent of colocalization of two proteins [24]. The Pearson’s correlation coefficient was significantly increased in *Arid5b*^*−/−*^ GC compared to *Arid5b*^+*/*+^ GC (*P* < 0.05) (Fig. 7b). These results indicate that there is enhanced GLUT4 translocation to the plasma membrane in *Arid5b*^*−/−*^ GC muscle.

To further confirm our results, we used primary skeletal muscle satellite cells isolated from *Arid5b*^+*/*+^ and *Arid5b*^*−/−*^ skeletal muscle [3]. These cells were differentiated for four days, and the assays were performed on day 4 myotubes. First, we evaluated the expression of TBC1D1, GLUT4, and GLUT1 in *Arid5b*^+*/*+^ and *Arid5b*^*−/−*^ primary day 4 myotubes. Similar to the *Arid5b*^*−/−*^ skeletal muscle, we found that only TBC1D1 expression was down-regulated in *Arid5b*^*−/−*^ primary myotubes compared to *Arid5b*^+*/*+^ myotubes while the expression level of GLUT4 and GLUT1 proteins was similar between the genotypes (Additional file 1: Fig. S7a). To confirm the increase in membrane GLUT4 content, we evaluated membrane GLUT4 in primary myotubes from each genotype using sulfo-NHS biotinylation method and immunoprecipitation of the biotinylated proteins with avidin beads, as detailed in the methods. We detected membrane and cytosolic GLUT4 and calculated the ratio of membrane GLUT4 content to cytosolic GLUT4 content. As shown in Additional file 1: Fig. S7b, the ratio was higher in *Arid5b*^*−/−*^ primary myotubes compared to *Arid5b*^+*/*+^ primary myotubes, suggesting an increase in GLUT4 membrane content. In addition, we conducted IHC for GLUT4 expression in GC muscle sections from mice of each genotype, and the result supports the increased membrane GLUT4 content in *Arid5b*^*−/−*^ skeletal muscle (Additional file 1: Fig. S7c). Collectively, these results are in agreement with the coimmunoflurescence data suggesting that the elevated basal glucose uptake in *Arid5b*^*−/−*^ skeletal muscle could be due to the downregulation of TBC1D1, which leads to an increase in translocation of GLUT4 to the cell membrane.

## Discussion

In this study, we characterized skeletal muscle metabolism in *Arid5b*^*−/−*^ mice and showed that glucose metabolism was upregulated in *Arid5b*^*−/−*^skeletal muscle. Our results suggest that the enhancement of glucose clearance in *Arid5b*^*−/−*^ mice and glucose uptake in skeletal muscle was insulin-independent and associated with the downregulation of TBC1D1, an inhibitor of GLUT4 translocation.

In *Arid5b*^*−/−*^ skeletal muscle, we observed increases in oxygen consumption, glucose oxidation, and ATP content. While these metabolic changes can be associated with morphological changes to the mitochondria, we did not observe alterations in mitochondrial morphology. Also, the expression of factors regulating mitochondrial biogenesis, such as PGC-1α, or factors regulating fusion and fission of mitochondria remained unchanged. Moreover, we did not observe changes in AMPK phosphorylation levels or the expression of Myh isoforms. Further investigation is necessary to elucidate the mechanism of the increased ATP content in *Arid5b*^*−/−*^ skeletal muscle. Given the increased glucose uptake rate in *Arid5b*^*−/−*^ mice shown by in vivo and in vitro studies, we hypothesized that the flux of glycolysis might be increased and might influence the rate of glucose oxidation in *Arid5b*^*−/−*^ muscle.

One of the important physiological roles of skeletal muscle is uptake of glucose from blood to normalize blood glucose concentration. Upon insulin stimuli, GLUT4 localizes to the plasma membrane to incorporate glucose into the cells. Perturbation of glucose uptake into skeletal muscle causes hyperglycemia, which eventually damages vessels and organs, such as kidney and heart [10]. Intracellular GLUT4 localization is controlled by small G protein Rabs, such as Rab8a and Rab14, which are physically associated with GLUT4 vesicles, and GTP-bound active Rabs are necessary to facilitate translocation of GLUT4 [25, 26]. TBC1D1 and TBC1D4 are Rab-GTPase-activating proteins (GAPs) that negatively control GLUT4 trafficking through their Rab-GAP activities under basal conditions [25, 27, 28]. Their activity is inhibited upon stimuli, such as insulin and exercise, through phosphorylation at multiple sites by Akt, AMPK, and Ca^2+^/calmodulin-dependent protein kinase kinase (CaMKK) [29]. Our results show an enhancement of glucose clearance in vivo and glucose uptake ex vivo in *Arid5b*^*−/−*^ mice without changes in plasma insulin levels or phosphorylation levels of Akt, which transduces signals from the insulin receptor to activate glucose transport. Instead of altering the insulin signaling pathway, the decreased expression of TBC1D1 in *Arid5b*^*−/−*^ skeletal muscle may influence glucose metabolism through GLUT4 translocation without altering GLUT4 expression. TBC1D1 downregulation has been shown to increase GLUT1 expression in cultured adipocytes [30]. However, no changes in GLUT1 expression were observed in *Arid5b*^*−/−*^ GC muscle, suggesting that the increased basal glucose uptake was not due to the induction of GLUT1 expression. Our coimmunofluroescence data demonstrated that GLUT4 localization to the plasma membrane was significantly increased in *Arid5b*^*−/−*^ skeletal muscle. These results suggest that enhanced GLUT4 translocation through TBC1D1 downregulation is the potential mechanism for the increased glucose uptake and oxidation in *Arid5b*^*−/−*^ skeletal muscle.

Our data are consistent with a previous report in L6 muscle cells showing that the downregulation of TBC1D1 increased basal GLUT4 translocation to the plasma membrane without altering GLUT4 expression [21]. In this regard, the downregulation of TBC1D1 has been reported in *Bmal1* skeletal muscle-specific knockout (*Bmal1*^*−/−*^) mice; however, insulin-stimulated glucose uptake but not basal glucose uptake was reduced in *Bmal1*^*−/−*^ skeletal muscle, which was accompanied by a decrease in GLUT4 protein expression [11]. Similarly, *Tbc1d1*-null mice showed a decrease in insulin-stimulated glucose uptake in skeletal muscle and reduced GLUT4 protein expression [23, 31]. In contrast, basal glucose uptake and the expression of GLUT4 were increased in skeletal muscle from mice with muscle-specific double knockout of insulin-like growth factor and insulin receptor (MIGIRKO) [22]. The discrepancy in glucose uptake in skeletal muscles from these knockout mice may be explained, at least partially, by the differences in GLUT4 protein levels. The potential mechanism for the regulation of GLUT4 protein levels by TBC1D1 remains to be established.

Moreover, in *Arid5b*^*−/−*^ whole skeletal muscle, we observed the downregulation of TBC1D1 at the protein level but not at mRNA level, suggesting that TBC1D1 protein level is decreased by translational regulation or reduced protein stability in *Arid5b*^*−/−*^ mice. However, we further analyzed *Tbc1d1* mRNA expression in primary myotubes from *Arid5b*^*−/−*^ and *Arid5b*^+*/*+^ mice, and found that *Tbc1d1* mRNA level was significantly decreased in *Arid5b*^*−/−*^ primary myotubes (Additional file 1: Fig. S8). Based on a Target Genes search in ChIP-Atlas for human ARID5B, *TBC1D1* is identified as a target gene of ARID5B (data not shown). These observations suggest that there is a possibility that TBC1D1 expression is regulated at mRNA level in myotubes from *Arid5b*^*−/−*^ mice.

Skeletal muscle is highly plastic and its proper metabolic functions are important for whole-body energy homeostasis [32]. Due to its high plasticity, skeletal muscle is sensitive to metabolic alterations in other tissues, such as adipose tissues and liver, and systemic factors, such as hormones and cytokines, all of which can affect the characteristics of skeletal muscle [8, 33]. In fact, in *Arid5b*^*−/−*^ mice, white adipose tissue mass was significantly reduced [5], and we cannot rule out the possibility that the characteristics of *Arid5b*^*−/−*^ skeletal muscle we showed in this report are non cell-autonomous. Nevertheless, this study provides additional information that the expression of TBC1D1 is critical for glucose uptake in skeletal muscle. The potential involvement of *Arid5b* in glucose metabolism through TBC1D1 would provide a new insight in the treatment of diabetes and obesity.

## Conclusions

Our results demonstrated that glucose metabolism is enhanced in the skeletal muscle from *Arid5b*^*−/−*^ mice, which was associated with the downregulation of TBC1D1. We provided additional information regarding the levels of TBC1D1 expression, glucose uptake, and GLUT4 expression in skeletal muscle. However, more research is necessary to elucidate the potential role of *Arid5b* in the regulation of TBC1D1 and GLUT4 expression.

## Electronic supplementary material

Below is the link to the electronic supplementary material.

**Additional file 1:** Additional figures.

## Data Availability

The datasets supporting the conclusions of this article are included within the article and its Additional file 1.

## Abbreviations

AMPK: AMP-activated protein kinase
ARID5B: AT-rich interactive domain 5b
*Arid5b*^*−*^^*/*^^*−*^: *Arid5b* knockout
*Arid5b*^+^^*/*^^+^: *Arid5b* wild-type
AUC: Area under the curve
*Bmal1*^*−*^^*/*^^*−*^: *Bmal1* skeletal muscle-specific knockout
BSA: Bovine serum albumin
CaMKK: Ca^2^^+^/calmodulin-dependent protein kinase kinase
Col2a1: Collagen type II alpha 1 chain
COX-1: Cyclooxygenase-1
CS: Citrate synthase
2-DG: 2-Deoxyglucose
DRP1: Dynamin-related protein 1
DTT: Dithiothreitol
EDL: Extensor digitorum longus
ETC: Electron transport chain
GAPs: Rab-GTPase-activating proteins
GC: Gastrocnemius
GLUT1: Glucose transporter 1
GLUT4: Glucose transporter 4
G6Pase: Glucose-6-phosphatase
HFD: High-fat diet
H3K9Me2: Dimethylated lysine 9 on histone 3
HSP70: Heat shock protein 70
HSP90: Heat shock protein 90
IHC: Immunohistochemistry
IP-GTT: Intraperitoneal glucose tolerance test
JARID: Jumonji AT-rich interactive domain
KHB: Krebs–Henseleit bicarbonate
MEFs: Mouse embryonic fibroblasts
MFN1: Mitofusin 1
MFN2: Mitofusin 2
MIGIRKO: Muscle-specific double knockout of insulin-like growth factor and insulin receptor
MSC: Mesenchymal stem cell
mTORC2: Mammalian target of rapamycin complex 2
MYH: Myosin heavy chain
OPA1: Optic atrophy 1
Pepck: Phosphoenolpyruvate carboxykinase
PGC-1α: Peroxisome proliferator-activated receptor gamma coactivator-1 alpha
PGI_2_: Prostaglandin I_2_
PHF2: Plant homeodomain finger protein 2
PKA: Protein kinase A
PMSF: Phenylmethylsulfonyl fluoride
PTGIS: Prostaglandin I_2_ synthase
QC: Quadriceps
qRT-PCR: Quantitative real-time PCR
Rpl13a: Ribosomal protein L13a
RT: Room temperature
SCs: Satellite cells
SDS: Sodium dodecyl sulfate
SoL: Soleus
SOX9: SRY-box transcription factor 9
TBC1D1: TBC1 domain family member 1
TBC1D4: TBC1 domain family member 4
TCA: Trichloroacetic acid
TNS: Tris-NaCl-Sucrose

## References

[1] Wilsker D, Probst L, Wain HM, Maltais L, Tucker PW, Moran E (2005). Nomenclature of the ARID family of DNA-binding proteins. Genomics.

[2] Yamakawa T, Whitson RH, Li S-L, Itakura K (2008). Modulator recognition factor-2 is required for adipogenesis in mouse embryo fibroblasts and 3T3-L1 cells. Mol Endocrinol.

[3] Murray J, Whitson RH, Itakura K (2018). Reduced prostaglandin I2 signaling in Arid5b−/− primary skeletal muscle cells attenuates myogenesis. FASEB J.

[4] Hata K, Takashima R, Amano K (2013). Arid5b facilitates chondrogenesis by recruiting the histone demethylase Phf2 to Sox9-regulated genes. Nat Commun.

[5] Whitson RH, Tsark W, Huang TH, Itakura K (2003). Neonatal mortality and leanness in mice lacking the ARID transcription factor Mrf-2. Biochem Biophys Res Commun.

[6] Yamakawa T, Sugimoto K, Whitson RH, Itakura K (2010). Modulator recognition factor-2 regulates triglyceride metabolism in adipocytes. Biochem Biophys Res Commun.

[7] Baba A, Ohtake F, Okuno Y (2011). PKA-dependent regulation of the histone lysine demethylase complex PHF2-ARID5B. Nat Cell Biol.

[8] Liesa M, Shirihai OS (2013). Mitochondrial dynamics in the regulation of nutrient utilization and energy expenditure. Cell Metab.

[9] Egan B, Zierath JR (2013). Exercise metabolism and the molecular regulation of skeletal muscle adaptation. Cell Metab.

[10] Kolluru GK, Bir SC, Kevil CG (2012). Endothelial dysfunction and diabetes: effects on angiogenesis, vascular remodeling, and wound healing. Int J Vasc Med.

[11] Dyar KA, Ciciliot S, Wright LE (2014). Muscle insulin sensitivity and glucose metabolism are controlled by the intrinsic muscle clock. Mol Metab.

[12] Mishra P, Carelli V, Manfredi G, Chan DC (2014). Proteolytic cleavage of Opa1 stimulates mitochondrial inner membrane fusion and couples fusion to oxidative phosphorylation. Cell Metab.

[13] Zanna C, Ghelli A, Porcelli AM (2008). OPA1 mutations associated with dominant optic atrophy impair oxidative phosphorylation and mitochondrial fusion. Brain.

[14] Duvezin-Caubet S, Jagasia R, Wagener J (2006). Proteolytic processing of OPA1 links mitochondrial dysfunction to alterations in mitochondrial morphology. J Biol Chem.

[15] Mai S, Klinkenberg M, Auburger G, Bereiter-Hahn J, Jendrach M (2010). Decreased expression of Drp1 and Fis1 mediates mitochondrial elongation in senescent cells and enhances resistance to oxidative stress through PINK1. J Cell Sci.

[16] Duguez S, Féasson L, Denis C, Freyssenet D (2002). Mitochondrial biogenesis during skeletal muscle regeneration. Am J Physiol Endocrinol Metab.

[17] Leary SC, Battersby BJ, Hansford RG, Moyes CD (1998). Interactions between bioenergetics and mitochondrial biogenesis. Biochim Biophys Acta Bioenerg.

[18] Larsen S, Nielsen J, Hansen CN (2012). Biomarkers of mitochondrial content in skeletal muscle of healthy young human subjects. J Physiol.

[19] Ren JM, Barucci N, Marshall BA, Hansen P, Mueckler MM, Shulman GI (2000). Transgenic mice overexpressing GLUT-1 protein in muscle exhibit increased muscle glycogenesis after exercise. Am J Physiol Endocrinol Metab.

[20] Liu P, Gan W, Chin YR (2015). PtdIns(3,4,5)P3-dependent activation of the mTORC2 kinase complex. Cancer Discov.

[21] Ishikura S, Klip A (2008). Muscle cells engage Rab8A and myosin Vb in insulin-dependent GLUT4 translocation. Am J Physiol Cell Physiol.

[22] O’Neill BT, Lauritzen HPMM, Hirshman MF, Smyth G, Goodyear LJ, Kahn CR (2015). Differential role of Insulin/IGF-1 receptor signaling in muscle growth and glucose homeostasis. Cell Rep.

[23] Szekeres F, Chadt A, Tom RZ, Deshmukh AS, Chibalin AV, Björnholm M, Al-Hasani H, Zierath JR (2012). The Rab-GTPase-activating protein TBC1D1 regulates skeletal muscle glucose metabolism. Am J Physiol - Endocrinol Metab.

[24] Dunn KW, Kamocka MM, McDonald JH (2011). A practical guide to evaluating colocalization in biological microscopy. Am J Physiol - Cell Physiol.

[25] Sano H, Kane S, Sano E, Mîinea CP, Asara JM, Lane WS, Garner CW, Lienhard GE (2003). Insulin-stimulated phosphorylation of a Rab GTPase-activating protein regulates GLUT4 translocation. J Biol Chem.

[26] Tunduguru R, Thurmond DC (2017). Promoting glucose transporter-4 vesicle trafficking along cytoskeletal tracks: PAK-ing them out. Front Endocrinol (Lausanne).

[27] Roach WG, Chavez JA, Mîinea CP, Lienhard GE (2007). Substrate specificity and effect on GLUT4 translocation of the Rab GTPase-activating protein Tbc1d1. Biochem J.

[28] Sakamoto K, Holman GD (2008). Emerging role for AS160/TBC1D4 and TBC1D1 in the regulation of GLUT4 traffic. Am J Physiol Endocrinol Metab.

[29] Cartee GD (2015). Roles of TBC1D1 and TBC1D4 in insulin- and exercise-stimulated glucose transport of skeletal muscle. Diabetologia.

[30] Zhou QL, Jiang ZY, Holik J, Chawla A, Hagan GN, Leszyk J, Czech MP (2008). Akt substrate TBC1D1 regulates GLUT1 expression through the mTOR pathway in 3T3-L1 adipocytes. Biochem J.

[31] Dokas J, Chadt A, Nolden T, Himmelbauer H, Zierath JR, Joost HG, Al-Hasani H (2013). Conventional knockout of Tbc1d1 in mice impairs insulin- and AICAR-stimulated glucose uptake in skeletal muscle. Endocrinology.

[32] Argilés JM, Campos N, Lopez-Pedrosa JM, Rueda R, Rodriguez-Mañas L (2016). Skeletal muscle regulates metabolism via interorgan crosstalk: roles in health and disease. J Am Med Dir Assoc.

[33] Meier U, Gressner AM (2004). Endocrine regulation of energy metabolism: review of pathobiochemical and clinical chemical aspects of leptin, ghrelin, adiponectin, and resistin. Clin Chem.

