# Correction to: Increased glucose metabolism in *Arid5b*^*−/−*^ skeletal muscle is associated with the down-regulation of TBC1 domain family member 1 (TBC1D1)

**DOI:** 10.1186/s40659-022-00371-9

**Published:** 2022-02-03

**Authors:** Yuri Okazaki, Jennifer Murray, Ali Ehsani, Jessica Clark, Robert H. Whitson, Lisa Hirose, Noriyuki Yanaka, Keiichi Itakura

**Affiliations:** 1 Department of Molecular and Cellular Biology, Beckman Research Institute, City of Hope, Duarte, CA USA; 2 Graduate School of Biosphere Science, Hiroshima University, Higashi-Hiroshima, Japan; 3 Department of Central Research Institute, Wakunaga Pharmaceutical Co., Ltd., Akitakata, Hiroshima Japan

## Correction to: Biol Res (2020) 53:45 https://doi.org/10.1186/s40659-020-00313-3

Following publication of the original article [1], the authors identified a mistake in the acknowledgement section.

- The correct version of acknowledgement is given below,
- The grant number 8 P20 GM103527-06 was reported in error. The authors would like to remove this grant number from the manuscript. All core facilities used for this research were supported by the National Cancer Institute of the National Institutes of Health under grant number P30 CA033572.
