# Supplementary material for: Increased glucose metabolism in Arid5b−/− skeletal muscle is associated with the down-regulation of TBC1 domain family member 1 (TBC1D1)
Source: Biol Res. 2020 Oct 6;53:45. doi: 10.1186/s40659-020-00313-3 (PMC7542134; doi:10.1186/s40659-020-00313-3)
Supplement: Supplementary file 1 — Additional file 1: Additional figures. [file 40659_2020_313_MOESM1_ESM.pdf]

Increased glucose metabolism in *Arid5b*$^{-/-}$ skeletal muscle is associated with the down-regulation of TBC1 domain family member 1 (TBC1D1)

Yuri Okazaki, Jennifer Murray, Ali Ehsani, Jessica Clark, Robert H. Whitson, Lisa Hirose, Noriyuki Yanaka, and Keiichi Itakura

Supplementary Figures:

Figure S1. Analysis of ADP and AMP contents

Figure S2. Analysis of protein expression of mitochondrial fusion and fission factors.

Figure S3. Protein expression of five ETC subunits in GC muscles.

Figure S4. mRNA expression levels of Myh isoforms.

Figure S5. Analysis of expression and phosphorylation levels of AKT.

Figure S6. Analysis of *Tbc1d1* and *Tbc1d4* mRNA expression in GC muscles.

Figure S7. TBC1D1 expression and detection of membrane GLUT4 in primary myotubes and immunohistochemistry (IHC) for GLUT4 in GC muscles.

Figure S8. Analysis of *Tbc1d1* mRNA expression in primary myotubes.

Supplementary Materials and Methods:

Nucleotide extraction

Antibodies for western blotting analysis

Primer sequences for quantitative real-time PCR (qRT-PCR)

IHC

Cell culture

Detection of membrane GLUT4 content

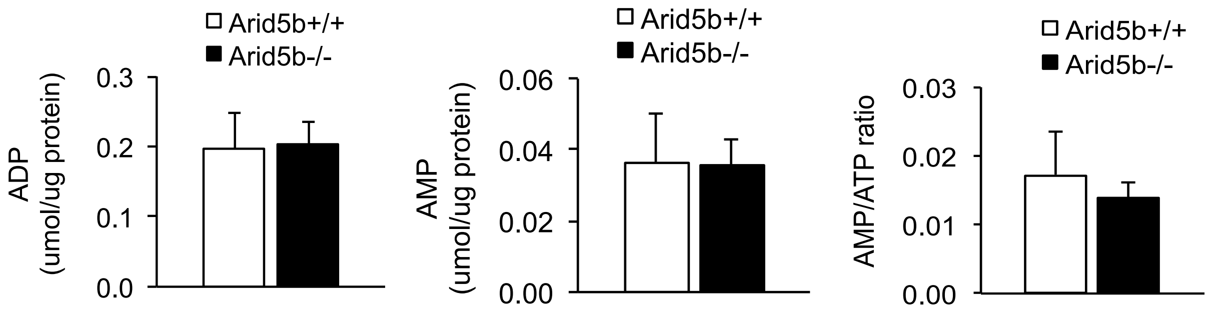

Fig. S1. Analysis of ADP and AMP contents. ADP, and AMP content were analyzed and AMP/ATP ratio was calculated in GC muscles. *Arid5b*$^{+/+}$ mice (n=11) and *Arid5b*$^{-/-}$ mice (n=6). Data are presented as means ± SD.

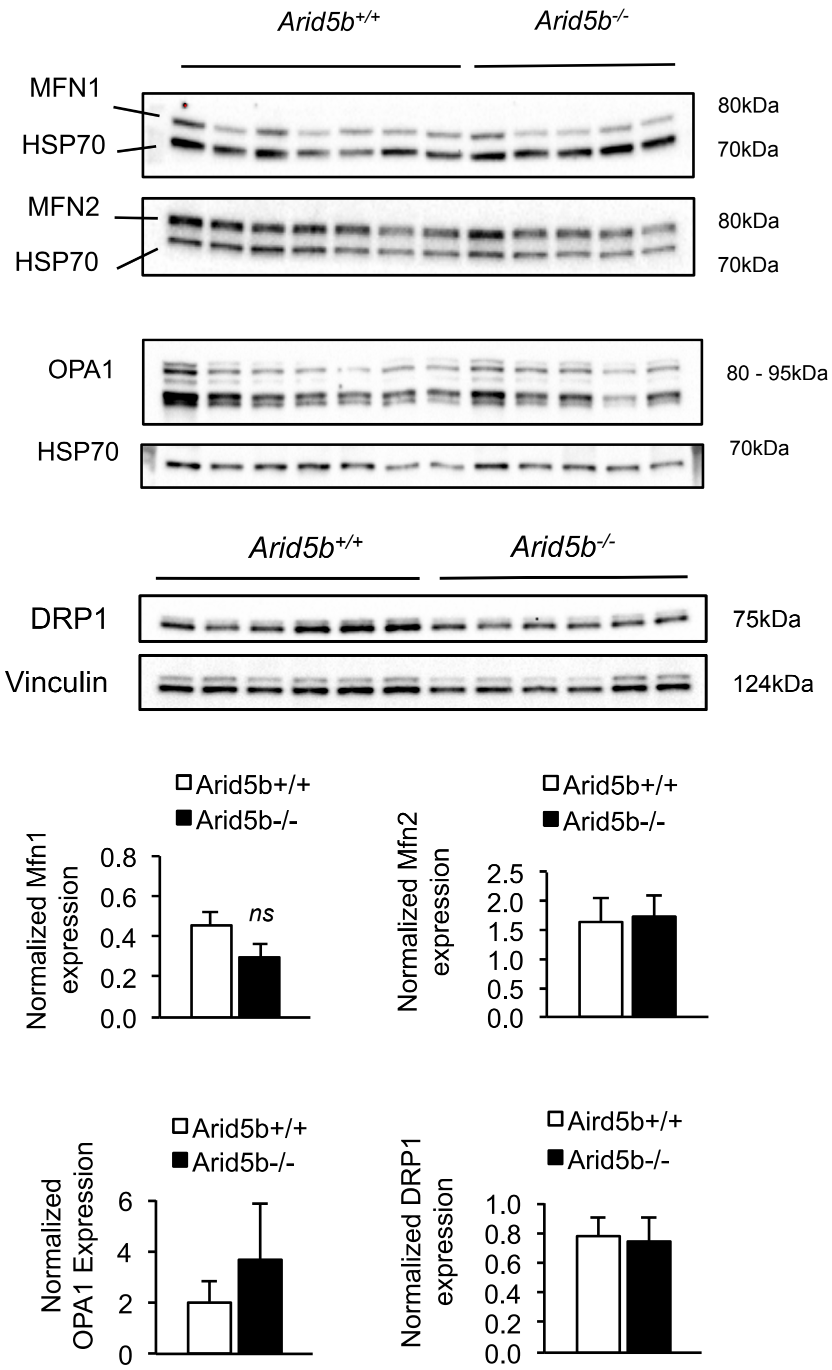

Fig. S2. Analysis of protein expression of mitochondrial fusion and fission factors. Protein expression of MFN1, MFN2, OPA1, DRP1 in the lysates prepared from GC muscle is shown. MFN1 and HSP70, and MFN2 and HSP70, were detected in the same membranes. Quantitation of protein expression to the corresponding loading control was performed, and data are presented as means ± SD (n=5-7).

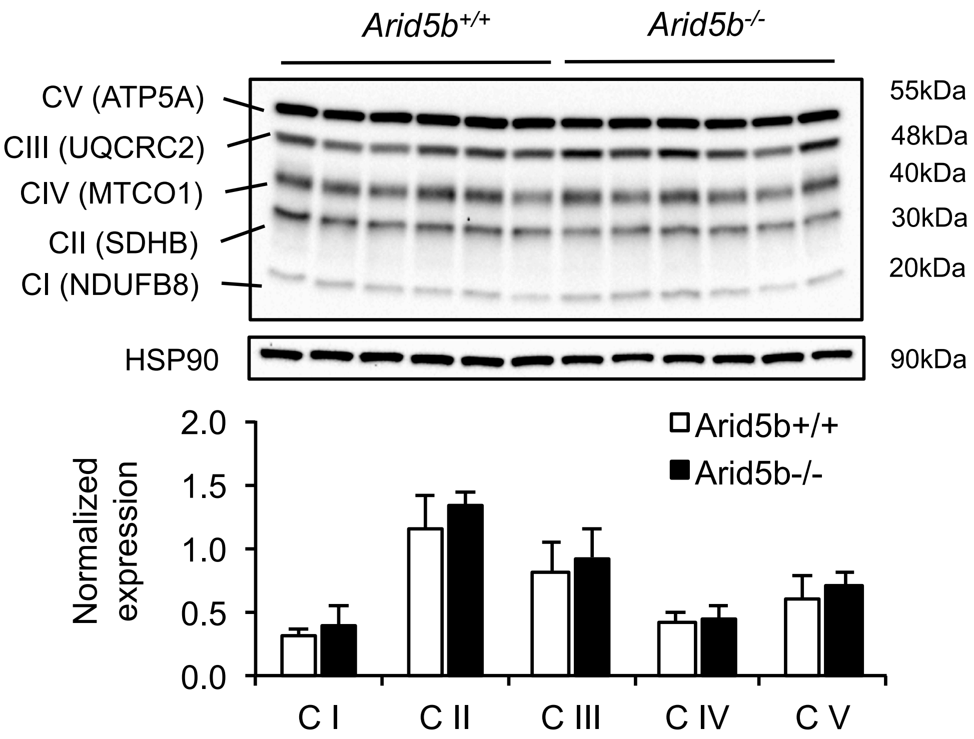

Fig. S3. Protein expression of five ETC subunits in GC muscles. Protein expression of each ETC subunit was normalized to the loading control (HSP90). CI: NADH: Ubiquinone oxidoreductase subunit B8 (NDUFB8), CII: succinate dehydrogenase complex iron sulfur subunit B (SDHB), CIII: ubiquinol-cytochrome C reductase core protein 2 (UQCRC2), CIV: mitochondrially encoded cytochrome C oxidase I (MTCO1), CV: ATP synthase, H+ transporting, mitochondrial F1 complex, alpha subunit 1 (ATP5A1). Immunoblot data is shown at the top, and a graph showing the normalized protein expression of five subunits in the electron transport chain is shown at the bottom. Data are presented as the means ± SD (n=6).

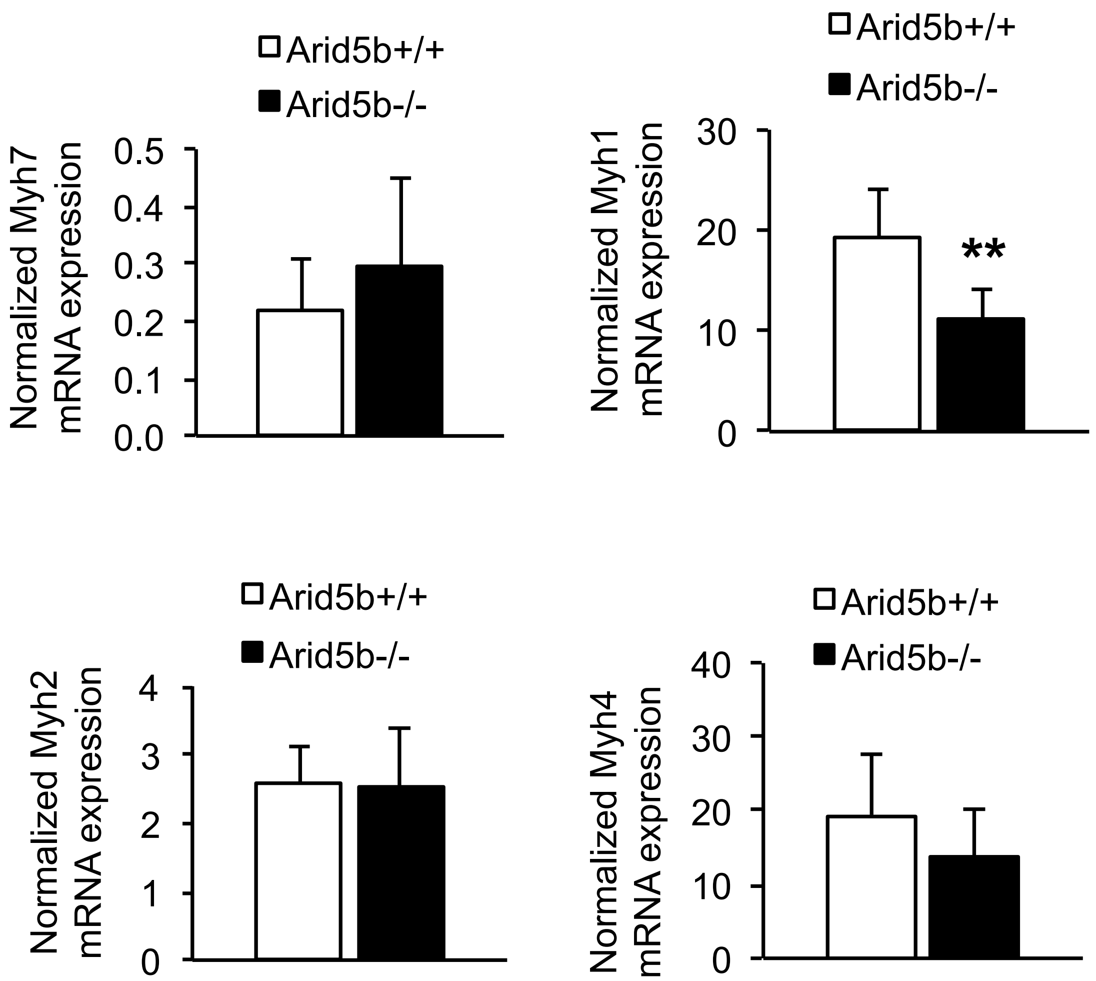

Fig. S4. mRNA expression levels of Myh isoforms. Total RNA was isolated from GC muscle, and qRT-PCR analysis was performed for the indicated Myh isoforms. Gene expression was normalized to *Rpl13a* mRNA expression. Data are presented as means ± SD (n=5-8). **, $P < 0.01$.

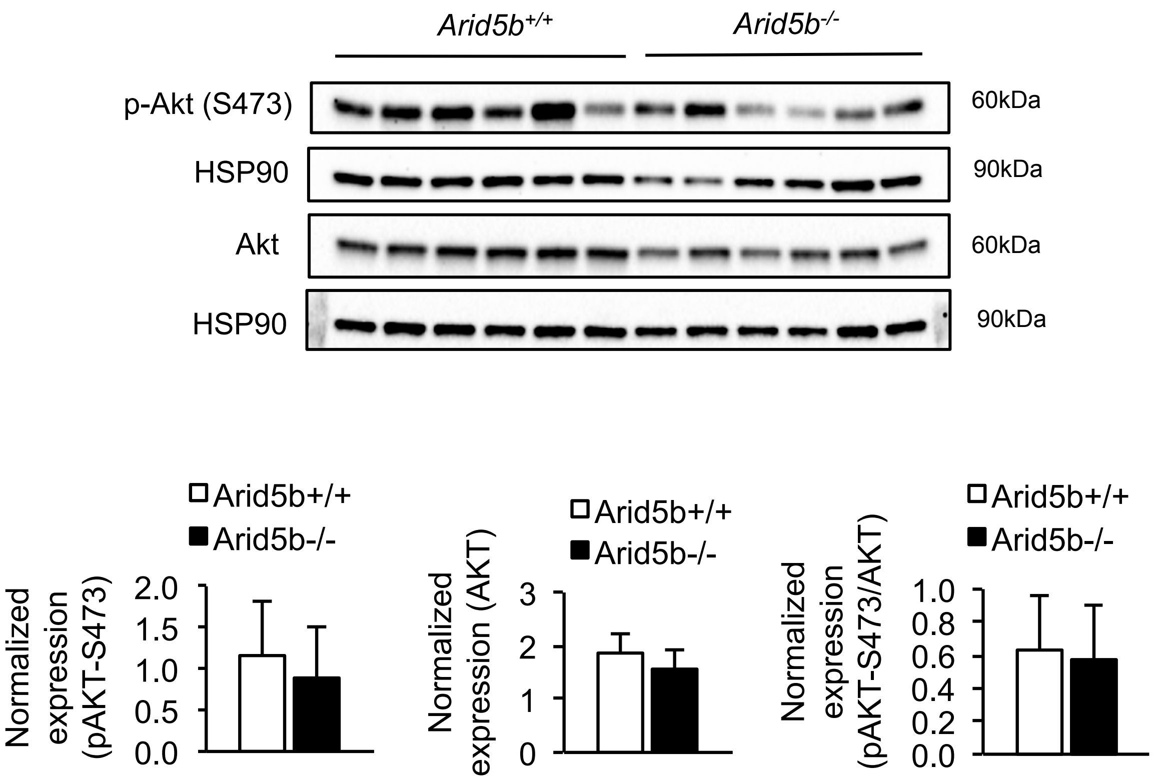

Fig. S5. Analysis of expression and phosphorylation levels of AKT. Western analysis was performed for Akt S473 phosphorylation levels (top) and total Akt expression. (bottom) Quantitation of p-Akt vs. Akt was carried out, and the graphs represent the means ± SD (n=6).

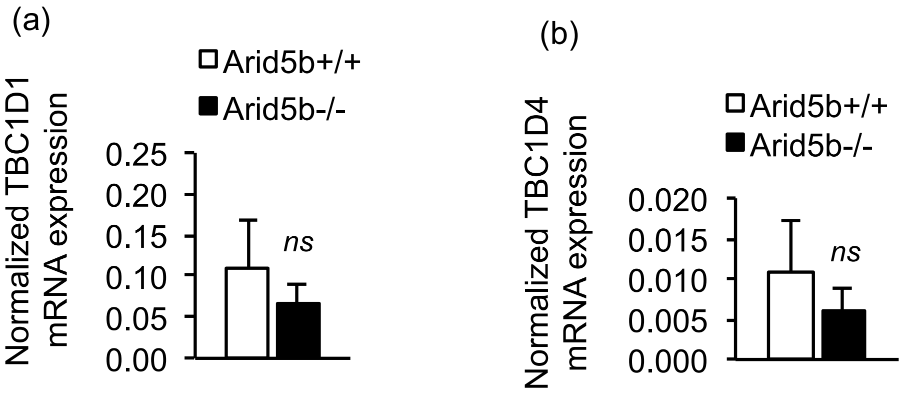

Fig. S6. Analysis of *Tbc1d1* and *Tbc1d4* mRNA expression in GC muscles. (a) *Tbc1d1* mRNA and (b) *Tbc1d4* mRNA are shown and data are presented as means ± SD (n=5-7).

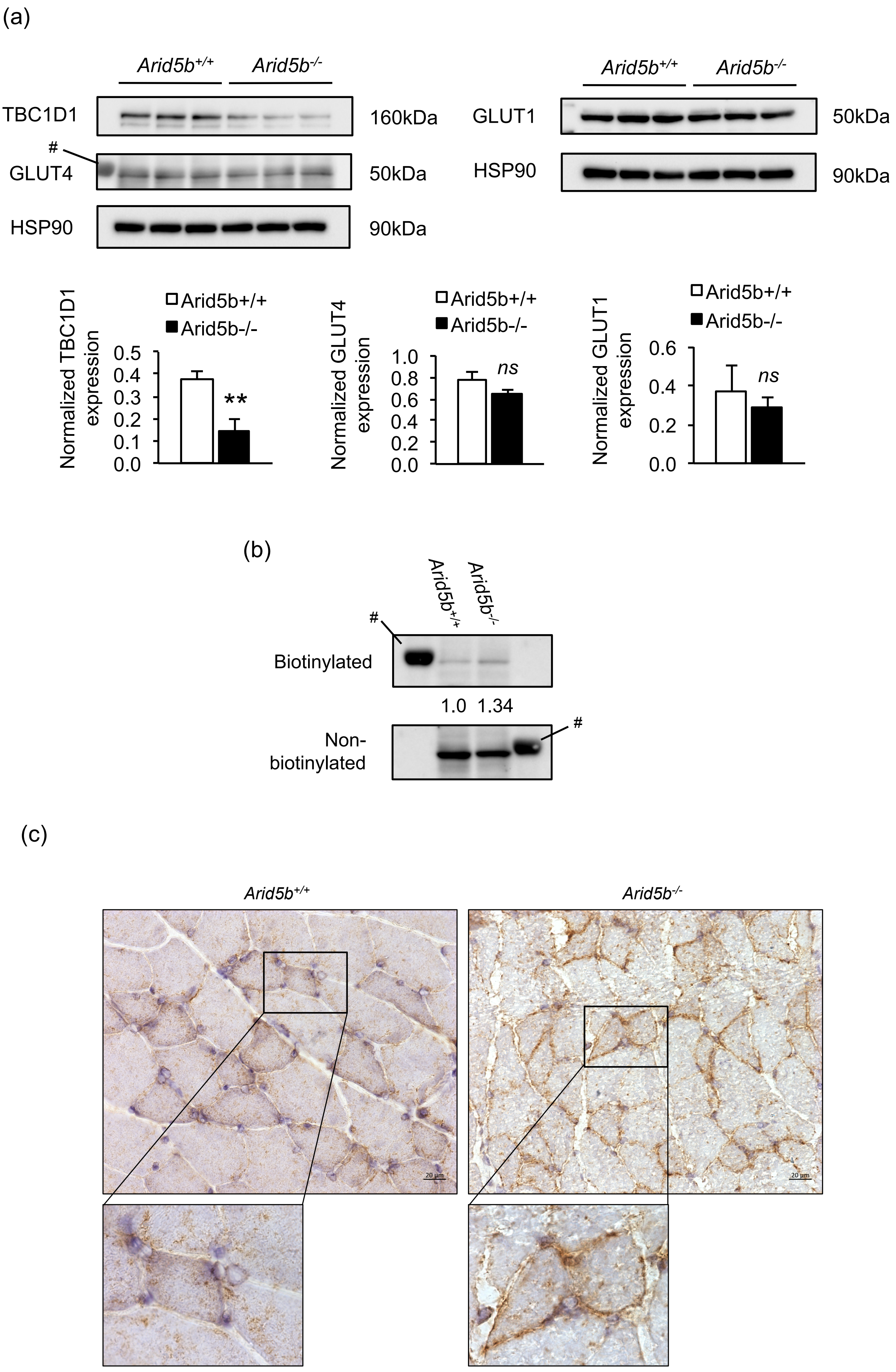
(a)
Arid5b+/+
Arid5b-/-
TBC1D1
160kDa
#
GLUT4
50kDa
HSP90
90kDa
GLUT1
50kDa
HSP90
90kDa
Normalized TBC1D1 expression
Arid5b+/+
Arid5b-/-
**
0.0
0.1
0.2
0.3
0.4
0.5
Normalized GLUT4 expression
ns
0.0
0.2
0.4
0.6
0.8
1.0
Normalized GLUT1 expression
ns
0.0
0.2
0.4
0.6
(b)
Arid5b+/+
Arid5b-/-
#
Biotinylated
1.0
1.34
Non-biotinylated
#
(c)
Arid5b+/+
Arid5b-/-
20 µm
20 µm

Fig. S7. TBC1D1 expression and detection of membrane GLUT4 in primary myotubes and IHC for GLUT4 in GC muscles. (a) The expression of TBC1D1, GLUT4, and GLUT1 was analyzed in primary myotubes at day 4 of differentiation (n=3). The expression levels of each protein were normalized to HSP90. #; marker. (b) Analysis of membrane GLUT4 at day 4 of differentiation. Membrane and cytosolic GLUT4 were immunoprecipitated using the method of protein biotinylation using sulfo-NHS biotin and collection of biotinylated proteins by avidin beads. The ratio of biotinylated fraction (membrane GLUT4) to non-biotinylated fraction (cytosolic GLUT4) was calculated. #; marker. **, $P < 0.01$. (c) IHC for GLUT4. Representative images of GLUT4 IHC (brown) in GC muscles counterstained with haematoxylin are shown.

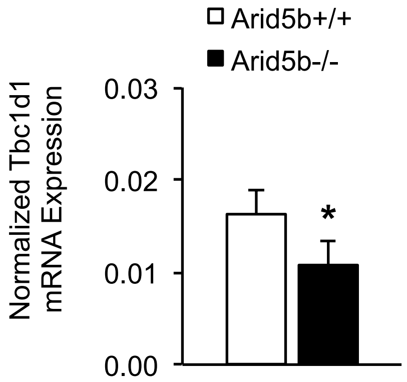

Fig. S8. Analysis of *Tbc1d1* mRNA expression in primary myotubes. *Tbc1d1* mRNA is shown and data are presented as means ± SD (n=4). *, $P < 0.05$.

Supplementary Materials and Methods

Nucleotide extraction

The details procedures of nucleotide extraction from GC muscles are shown in the main manuscript.

Antibodies for western blotting analysis

The details procedures of western blotting analysis are shown in the main manuscript. RIPA lysis buffer (50 mM Tris-HCL, 150 mM NaCl, 0.25% deoxycholic acid, 1% NP-40, 1 mM EDTA, 0.1% SDS [pH 7.4]) was used to prepare lysates from primary myotubes. The following antibodies were used for the analysis: anti-MFN1 antibody (Abcam, # ab126575), anti-MFN2 antibody (Cell Signaling, #9482), anti-OPA1 antibody (Abcam, # ab42364), anti-DRP1 antibody (Cell Signaling, #5391), total OXPHOS rodent western blot antibody cocktail (Abcam, #ab110413), anti-phospho-Akt (Ser473, Cell Signaling, #4060), anti-Akt (Cell Signaling, #9272), anti-GLUT4 (Cell Signaling, #2213), anti-GLUT1 (Novus Biologicals, #NB110-39113), anti-TBC1D1 (Cell Signaling, #4629), anti-HSP70 antibody (Cell Signaling, #4872), anti-HSP90 antibody (Cell Signaling, #4877), and anti-vinculin (Cell Signaling, #13901). Western BLoT Immuno Booster (Takara, #T7111A) was used to incubate antibodies for the detection of p-Akt (Ser473), GLUT4 and TBC1D1.

Primer sequences for quantitative real-time PCR (qRT-PCR)

The details procedures of qRT-PCR are shown in the main manuscript. The primers shown in Table S1 were used for qRT-PCR.

Table S1.

Gene symbol	Forward (5' – 3')	Reverse (5' – 3')
Myh1	TTGACTTTGGGATGGACCTG	TCTTGAAGGAGGTGTCTGTC
Myh2	AGGACCAAATCATCAGTGCC	TGTCAGCAGATGCCAGTTTC
Myh4	TTGATGACCAGGAAGAGCTG	ATAATGCATCACAGCGCCTG
Myh7	TGGAGAATGACAAGCAGCAG	TTGCTCATCCTCAATCCTGG
Tbc1d1	TACCTCATCAGTCCTGACAC	AGTGGTCCACATGTCTGATG
Tbc1d4	TCTTTGCCTCTCAGTTTCCC	CTCAGTAAGCTGAGAGCAAC

IHC

Skeletal muscle tissue was dissected from mice, placed on corkboard (2cm x 2cm) using tragacanth gum and immediately frozen in isopentane that was cooled with liquid nitrogen. Cryosections (10 μm thick) were placed on positively charged glass slides. Sections were dried at room temperature, fixed in acetone for 10 min, and treated with 3% hydrogen

peroxide for 5 min to quench endogenous peroxidase activity. Sections were then incubated with anti-GLUT4 antibody (Abcam, #ab33780) followed by incubation with EnVision+ System-HRP labeled polymer anti-rabbit (DAKO) and Liquid DAB+ Substrate Chromogen System (DAKO). After washing, the sections were counterstained with haemotoxylin and coverslipped. Images were taken with the VENTANA iScan HT slide scanner (Ventana). The myofibers that show GLUT4 staining near cell membrane were counted in GC and the percentage of them was calculated. The average number of fibers counted per GC was about 1,200.

Cell culture

Primary skeletal muscle satellite cells were isolated from mouse hindlimb skeletal muscle previously, and the isolation method was shown in our previous report [1]. Primary muscle satellite cells were cultured in growth media containing 40% DMEM, 40% Ham's F-10, 20% fetal bovine serum, 100 U/mL penicillin, and 100 µg/mL streptmycin, and 2.5 ng/mL basic-FGF. Myogenic differentiation was induced with differentiation medium (DM) containing DMEM with 5% horse serum, 100 U/mL penicillin, and 100 µg/mL streptmycin for four days. DM was changed every day.

Detection of membrane GLUT4 content

Primary myotubes at day 4 of differentiation were used for the assay. We adapted the detection method from Llanos et al. [2] and modified it for the assay with primary myotubes. Briefly, the differentiated myotubes were incubated with 0.5 mg/mL of sulfo-NHS biotin (Thermo scientific, #21217) for 1 hour after washed twice with PBS. After 1 hour, the reaction was stopped by adding 100 mM glucine and the cells were washed twice with PBS. The cells were harvested with cold lysis buffer (140 mM NaCl, 1% Triton X-100, 1 mM EDTA, 1 mM EGTA, and 20 mM Tris-HCl, pH 7.5, 1 x Halt protease and phosphatase inhibitor) and homogenized by sonication in an ice-water bath. The lysates were incubated on ice for 30 min and then centrifuged at 3,000 x *g* for 30 min at 4°C. The supernatant was collected, and protein concentration was determined with the Pierce™ BCA assay kit. Protein concentration was adjusted and same amount of the lysate was incubated overnight with NeutrAvidin plus ultralink resin (Thermo Scientific, #53151) under constant rotation. The next day, the non-biotinylated fraction in the supernatant was collected by centrifugation at 14,000 rpm for 15 min at 4°C. The biotinylated fraction that remained in the pellet was washed twice with lysis buffer, re-suspended in 1 x Sample buffer for SDS-PAGE, and incubated at 65°C for 30 min. The biotinylated fraction in the supernatant was separated from beads by centrifugation at 14,000 rpm for 2min. The GLUT4 protein content in the biotinylated fraction (plasma membrane fraction) and in the non-biotinylated fraction (cytosolic fraction) was detected by western blotting.

References

1. Murray J, Whitson RH, Itakura K (2018) Reduced prostaglandin I2 signaling in Arid5b-/- primary skeletal muscle cells attenuates myogenesis. FASEB J 32:1868–1879
2. Llanos P, Ferrat-Contreras A, Georgiev T, Osorio-Fuentealba C, Espinosa A, Hidalgo J, Hidalgo C, Jaimovich E (2015) The cholesterol-lowering agent methyl-β-cyclodextrin promotes glucose uptake via GLUT4 in adult muscle fibers and reduces insulin resistance in obese mice. Am J Physiol - Endocrinol Metab 308:E294–E305
